# Rapid Ocular Responses Are Modulated by Bottom-up-Driven Auditory Salience

**DOI:** 10.1523/JNEUROSCI.0776-19.2019

**Published:** 2019-09-25

**Authors:** Sijia Zhao, Nga Wai Yum, Lucas Benjamin, Elia Benhamou, Makoto Yoneya, Shigeto Furukawa, Fred Dick, Malcolm Slaney, Maria Chait

**Affiliations:** ^1^Ear Institute, University College London, London WC1X 8EE, United Kingdom,; ^2^Dementia Research Centre, Department of Neurodegenerative Disease, University College London, London WC1N 3AR, United Kingdom,; ^3^NTT Communication Science Laboratories, NTT Corporation, Atsugi 243-0198 Japan,; ^4^Department of Psychological Sciences, Birkbeck College, London WC1 7HX, United Kingdom,; ^5^ Department of Experimental Psychology, University College London, London WC1H 0DS, United Kingdom, and; ^6^Machine Hearing Research, Google, Mountain View, California 94043

**Keywords:** attention, auditory scene analysis, microsaccades, pupil dilation, superior colliculus

## Abstract

Despite the prevalent use of alerting sounds in alarms and human–machine interface systems and the long-hypothesized role of the auditory system as the brain's “early warning system,” we have only a rudimentary understanding of what determines auditory salience—the automatic attraction of attention by sound—and which brain mechanisms underlie this process. A major roadblock has been the lack of a robust, objective means of quantifying sound-driven attentional capture. Here we demonstrate that: (1) a reliable salience scale can be obtained from crowd-sourcing (*N* = 911), (2) acoustic roughness appears to be a driving feature behind this scaling, consistent with previous reports implicating roughness in the perceptual distinctiveness of sounds, and (3) crowd-sourced auditory salience correlates with objective autonomic measures. Specifically, we show that a salience ranking obtained from online raters correlated robustly with the superior colliculus-mediated ocular freezing response, microsaccadic inhibition (MSI), measured in naive, passively listening human participants (of either sex). More salient sounds evoked earlier and larger MSI, consistent with a faster orienting response. These results are consistent with the hypothesis that MSI reflects a general reorienting response that is evoked by potentially behaviorally important events regardless of their modality.

**SIGNIFICANCE STATEMENT** Microsaccades are small, rapid, fixational eye movements that are measurable with sensitive eye-tracking equipment. We reveal a novel, robust link between microsaccade dynamics and the subjective salience of brief sounds (salience rankings obtained from a large number of participants in an online experiment): Within 300 ms of sound onset, the eyes of naive, passively listening participants demonstrate different microsaccade patterns as a function of the sound's crowd-sourced salience. These results position the superior colliculus (hypothesized to underlie microsaccade generation) as an important brain area to investigate in the context of a putative multimodal salience hub. They also demonstrate an objective means for quantifying auditory salience.

## Introduction

Our perception of our surroundings is governed by a process of competition for limited resources. This involves an interplay between task-focused and bottom-up-driven processes that automatically bias perception toward certain aspects of the world, to which our brain, through experience or evolution, has been primed to assign particular significance. Understanding the neural processes that underlie such involuntary attentional capture is a topic of intense investigation in systems neuroscience (Itti and Koch, 2001, 2000; Kayser et al., 2005; Kaya and Elhilali, 2014).

Research in vision has capitalized on the fact that attentional allocation can be “objectively” decoded from ocular dynamics: Observers free-viewing complex visual scenes tend to demonstrate consistent fixation, saccade, and microsaccade patterns that can be used to infer the attributes that attract bottom-up visual attention (Parkhurst et al., 2002; Peters et al., 2005; Hafed et al., 2009; Yuval-Greenberg et al., 2014; Veale et al., 2017; Krauzlis et al., 2018). The underlying network for (micro-)saccade generation is centered on the superior colliculus (SC; Hafed et al., 2009) with a contribution from the frontal eye fields (Peel et al., 2016), consistent with a well established role for these regions in computing the visual salience map and controlling overt attention (Veale et al., 2017; White et al., 2017a,b).

The appearance of new events is also associated with two types of rapid orienting responses: (1) an “ocular freezing” (“microsaccadic inhibition”; MSI) response—a rapid transient decrease in the incidence of microsaccades, hypothesized to arise through suppression of ongoing activity in the SC by new sensory inputs (Hafed and Clark, 2002; Engbert and Kliegl, 2003; Rolfs et al., 2008; Hafed and Ignashchenkova, 2013); and (2) a phasic pupil dilation response (PDR; Wang and Munoz, 2015; Wang et al., 2017). The PDR has been linked to potentially SC-mediated (Wang and Munoz, 2015) spiking activity in the locus ceruleus (Joshi et al., 2016), which constitutes the source of norepinephrine (noradrenaline) to the central nervous system and therefore controls global vigilance and arousal.

Both MSI and PDR have been shown to systematically vary with visual salience (Rolfs et al., 2008; Bonneh et al., 2014; Wang and Munoz, 2014; Wang et al., 2017, 2014) and are theorized to reflect the operation of an interrupt process that halts ongoing activities so as to accelerate an attentional shift toward a potentially survival-critical event.

Interestingly, sounds can also drive these ocular responses. Abrupt, or otherwise out-of-context auditory events evoke pupil dilation, and cause MSI (Rolfs et al., 2005, 2008; Liao et al., 2016; Wetzel et al., 2016; Wang and Munoz, 2014, 2015; Wang et al., 2014), consistent with a proposal that these responses reflect the operation of a modality-general orienting mechanism. In fact, sounds cause faster responses than visual stimuli (Rolfs et al., 2008; Wang et al., 2014), consistent with the “early warning system” role of hearing (Murphy et al., 2013). However, because only very simple stimuli have been used, the degree to which sound-evoked ocular responses reflect acoustic properties beyond loudness (Liao et al., 2016; Huang and Elhilali, 2017) remains unknown.

Here, we sought to determine whether MSI and PDR are sensitive to auditory salience. We used crowd-sourcing to obtain a “subjective” (e.g., ratings-based) salience ranking of a set of brief environmental sounds. The obtained salience scale was also verified with a small, “in-laboratory” replication. Then in a laboratory setting, a group of naive participants passively listened to these sounds while their ocular dynamics and pupil dilation were recorded. We demonstrate that MSI (but not PDR) is systematically modulated by auditory salience. This is consistent with the hypothesis that MSI indexes a rapid, multimodal orienting mechanism that is sensitive to not just the onset, but also the specific perceptual distinctiveness of brief sounds.

## Materials and Methods

### 

#### Stimuli

**Figure 1.**
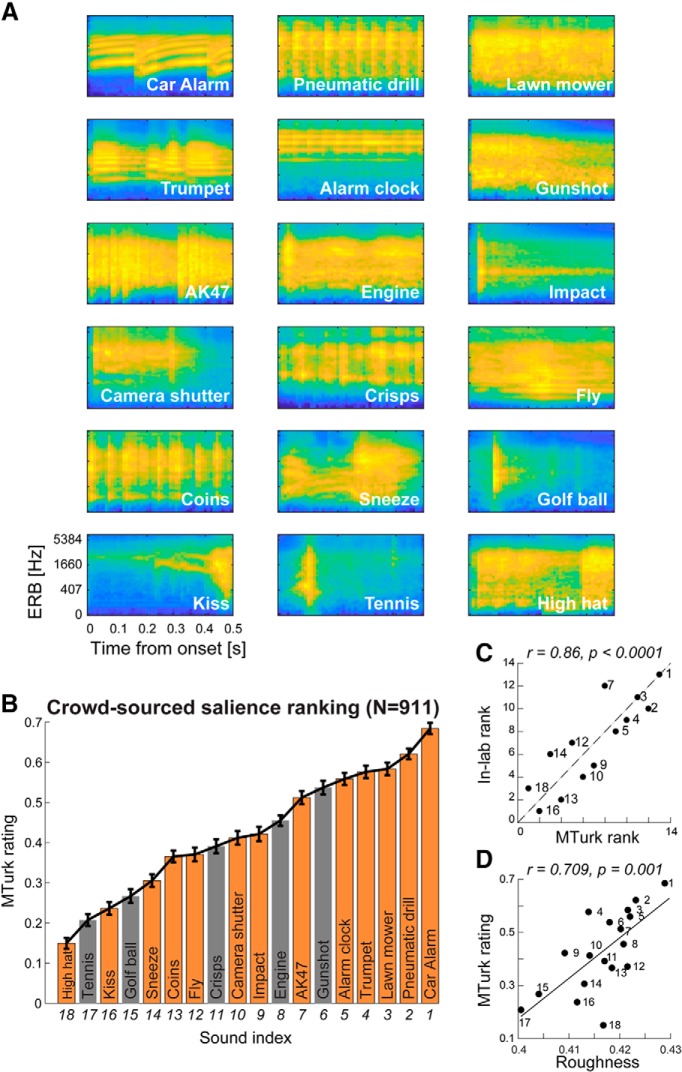
Crowd-sourced “subjective” salience rating for brief environmental sounds. ***A***, Spectrograms for all 18 sounds are displayed in order of ranking in ***B***. See Figure 1-1 in the extended data section, for sound files. ***B***, Crowd-sourced rating collected from MTurk (*N* = 911). The sounds used in the in-laboratory replication are indicated by orange-colored bars. Error bars are 1 SD from bootstrap resampling. See Figure 1-2 in the extended data section, for details of the instructions to online participants and MTurk page layout. ***C***, Crowd-sourced salience rating is strongly correlated with the in-laboratory salience ranking. The dashed line indicates identical ranks. ***D***, Crowd-sourced salience rating is strongly correlated with acoustic “roughness.” All correlations are conducted using the Spearman rank method.

Eighteen environmental sounds drawn from Cummings et al. (2006), with a subset from Dick and Bussiere (2002) and Saygin et al. (2005), were used. All stimuli were 500 ms long and RMS equated (see individual spectrograms in Fig. 1*A* and Fig. 1-1 in the extended data for sound files).

10.1523/JNEUROSCI.0776-19.2019.f1-1 Figure 1-1 Sound files for the stimuli used in this study. Download Figure 1-1, ZIP file

**Figure 2.**
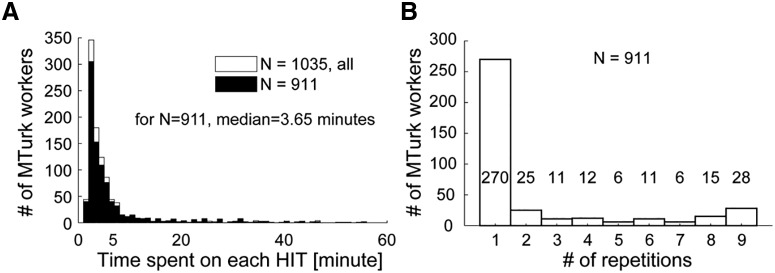
MTurk task data. ***A***, Distribution of time spent on each HIT. ***B***, Distribution of number of HITs completed per worker.

10.1523/JNEUROSCI.0776-19.2019.f1-2 Figure 1-2 An example of a HIT (‘human intelligence task’) page used in the crowd-sourcing experiment. Download Figure 1-2, TIF file

#### Subjective salience via crowd-sourcing

##### Experimental design and statistical analysis.

The experiment was conducted on the Amazon Mechanical Turk (MTurk) platform, an online marketplace that allows remote participants (“workers” per MTurk nomenclature) to perform tasks via a web interface. Raters were selected from a large pool of prequalified “workers” in the United States who were judged to be reliable over the course of many previous (non-salience-related) experiments. Each session is delivered through a “human intelligence task” (HIT) page, which contains instructions and the stimuli for that session (see Fig. 1-2 in the extended data section for an example of a HIT page used in the present experiment). Because we were interested in the extent to which a relatively free listening environment can result in meaningful data, we did not impose constraints on sound delivery (Woods et al., 2017) or level (though it was suggested that the participants listen over headphones).

Participants made relative salience judgments on sounds presented in pairs. In total, a pool of 7038 pairs (153 possible pairs × 2 orders to control for order effects × 23 repetitions) were generated. The pairs were then arranged in 207 different HITs by randomly selecting subsets of 34 different pairs from the above pool. Each HIT also included six randomly interspersed “catch trials” in which the two sounds were identical. Each HIT therefore contained 40 sound pairs along with task instructions. Each pair had its own “Play” button, which when pressed started the presentation of the corresponding sound pair, with a 500 ms silent gap between the two sounds. Participants were asked which sound was “more salient or noticeable.” “Which sound would you think is more distracting or catches your attention?” Participants could only listen to each pair once before responding by selecting one of the “first,” “second,” or “identical” buttons to progress to the next sound pair. Participants were instructed to choose the “identical” button only if the sounds were physically identical (catch trials). Failure to respond appropriately to the catch trials (or choosing the “identical” response for the noncatch trials) indicated lack of engagement with the experiment and resulted in the data from that session being excluded from analysis (∼10% exclusion rate, see below). Participants were offered financial compensation approximately equal to the minimum U.S. wage and prorated for the 5 min experiment time. To encourage participant engagement, we paid a small bonus when participants correctly responded to identical sounds and subtracted a small amount for each miss. Each HIT was run by five unique workers for an overall number of 1035 sessions. The time limit for task completion was set to 60 min, though we expected the experiment to last an average of 3 min. Figure 2*A* plots the actual duration distribution. Most sessions were completed within 3 min.

Each participant was free to complete up to a maximum of nine different HITs. A distribution of HITs per worker is in Figure 2*B*. Most (71.4%) completed one HIT only, whereas 52 workers (12.4%) completed the maximum number of HITs. We did not find any relationship between participants” number of HITs and performance on catch trials. From the total of 1035 sessions completed, 57 included a single missed catch trial, 11 included two missed catch trials and 11 included more than two missed catch trials. Fifty-eight sessions contained false positives. Overall, 124 sessions were excluded. The remaining sessions were composed of 384 unique workers, of which 270 completed only one HIT and the rest completed multiple HITs.

Salience ranking was computed by pooling across all HITs and counting the proportion of pairs on which each sound was judged as more salient. Variability was estimated using bootstrap resampling (1000 iterations), where on each iteration, one session for each of the 207 unique HITs was randomly selected for the ranking analysis. The error bars in Figure 1*B* are one standard deviation from this analysis. The same ranking was obtained after removing sessions with durations exceeding the 90th percentile (14.09 min, *n* = 820 remaining) or the 75th percentile (5.98 min, *n* = 683 remaining).

##### Acoustic analysis.

The salience data were analyzed to examine possible correlations with several key acoustic features previously implicated in perceptual salience.

An overall loudness measure was produced by a model in which the acoustic signal was filtered with a bank of bandpass filters of width 1 ERB (Moore and Glasberg, 1983) and center frequencies spaced 1/2 ERB from 30 Hz to 16 kHz. The instantaneous power of each filter output was smoothed with a 20 ms window and elevated to power 0.3 to approximate specific loudness (Hartmann, 1996). Outputs were then averaged across channels to produce a single value. This model was preceded by a combination of high-pass and low-pass filters to approximate effects of outer and middle ear filtering (Killion, 1978).

Several key measures of salience derived from the model of Kayser et al. (2005) were examined. Paralleling work in the visual modality (Itti and Koch, 2001), this model produces an auditory saliency map in the form of a frequency x time representation, indicating the spectrotemporal loci that are hypothesized to be particularly perceptually salient. The representation is computed by independently extracting several key auditory features (loudness, spectral and temporal contrast), which are normalized to create a feature-independent scale and then linearly combined together to create the overall map. For the present analysis, we extracted several parameters from the salience map computed for each sound-token: the maximum value within the saliency map (this is the parameter used in the experiments reported in Kayser et al., 2005), the mean saliency value across the entire map, and max/mean gradient across the frequency and/or time dimensions.

**Figure 3.**
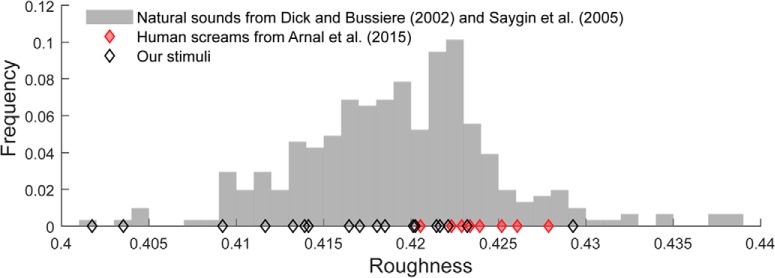
To estimate the range of “roughness” values that we might expect to encounter in the environment, we quantified roughness (see Materials and Methods) for a large set of diverse natural sounds (*N* = 274; sound duration = 500 ms to match that in the present experiment) from a set described previously (Dick and Bussiere, 2002; Saygin et al., 2005). This information is presented in histogram form (gray bars). Roughness values for the sounds used in the present study are indicated by black diamonds. We also include roughness calculated for the scream sounds from Arnal et al. (2015) (red diamonds). This analysis confirms that the set of sounds we used spans the range of roughness obtained from a diverse set of environmental sounds. The sounds at the top of the roughness range in our set, overlap with the roughness range defined by the scream sounds from Arnal et al. (2015).

Roughness was calculated from the modulation power spectrum, computed using the approach described in Elliott and Theunissen (2009) (see also Arnal et al., 2015). Roughness is associated with energy in the high end (>30 Hz) of the amplitude modulation spectrum, though it also depends on modulation depth and other spectral factors (Pressnitzer and McAdams, 1999). As is typical of natural wide-band sounds, in our sound set we found a strong correlation (Spearman *r* = 0.808, *p* < 0.0001) between power at high modulation rates (30–100 Hz) and those below 30 Hz (0–30 Hz). We also noted that salience (MTurk derived; see Results) significantly correlated with power at modulations between 30 and 100 Hz (Spearman, *r* = 0.585 *p* = 0.01) but not with the low frequency (0–30 Hz) modulations (Spearman *r* = 0.222 *p* = 0.376). Controlling for low frequency modulations as a covariate (partial correlation), yielded a substantially stronger correlation (Spearman *r* = 0.707 *p* = 0.002), suggesting that the high modulation rates, independently of overall modulation power, contributed to salience. Therefore, to specifically isolate the contribution of high modulation rates, and control for overall power across the modulation spectrum, “roughness” was quantified as the ratio between power at modulations between 30 and 100 Hz and power between 0 and 100 Hz (i.e., across the full range). See also Figure 3, for a comparison of how roughness in the present set relates to the range of roughness (calculated in the same way) obtained from a diverse set of environmental sounds.

#### Subjective salience via in-laboratory replication

##### Participants.

To verify that the online salience ranking data also hold when tested in a more controlled environment, 18 paid participants (15 females, average age 23.8, range 18–31) took part in an in-lab replication study; all reported normal hearing and no history of neurological disorders. Experimental procedures were approved by the research ethics committee of University College London and written informed consent was obtained from each participant.

##### Experimental design and statistical analysis.

We again used a pairwise task with identical presentation parameters as in the MTurk experiment, but that every participant was presented with the full set of all possible pairs (78 pairs × 2 possible orders × 2 repetitions) for a total of 312 pairs of sounds. These were presented in a random order in six consecutive blocks (∼8 min). Each block also contained eight randomly interspersed catch trials of identical sounds. Participants were allowed a short rest between blocks.

The stimuli were delivered to the participants” ears by Sennheiser HD558 headphones via a UA-33 sound card (Roland) at a comfortable listening level self-adjusted by each participant. Stimulus presentation and response recording were controlled with the Psychtoolbox package (Psychophysics Toolbox Version 3; Brainard, 1997) with MATLAB (The MathWorks). Participants were tested in a darkened and acoustically shielded room (IAC triple-walled sound-attenuating booth). The session lasted for 1 h, starting with the same instructions given in the MTurk experiment. Participants were instructed to fixate their gaze on a white cross at the center of the computer screen while listening to the stimuli, and to respond by pressing one of 3 keyboard buttons to indicate “sound A more salient”/“sound B more salient”/“identical sounds.” The participant's response initiated the following trial with a random intertrial interval of 1.5 to 2 s. Blocks featuring incorrect responses—whether a miss or a false alarm—to any of the eight catch trials indicated lack of engagement and the whole block was discarded from the analysis. In this instance, all participants performed perfectly with a 100% hit rate and 0% false alarm rate resulting in no exclusions.

#### Eye tracking

##### Participants.

This experiment was performed by a total of 30 paid participants (28 females; aged 18–29, average 23.33), with 15 participants initially (14 females; aged 21∼28, average 23.53), subsequently supplemented by an additional group of 15 participants so as to have a better measure of variability across the population. No participants were excluded from this experiment. All reported normal hearing and no history of neurological disorders. All participants were naive to the aims of the experiment and none had participated in the in-laboratory ranking experiment described above. Experimental procedures were approved by the research ethics committee of University College London and written informed consent was obtained from each participant.

##### Experimental design and statistical analysis.

Sixteen sounds out of the original set were used in this experiment. Sound #3 and sound #9 (Fig. 1) were excluded due to experiment length constraints.

The effective onset (the time point to which the eye tracking analysis is time locked) was adjusted for each sound-token to a time where level exceeded a fixed threshold. The threshold was defined as the 20^th^ percentile of the distribution of power (per time sample; over the initial 50 ms) pooled across sound tokens. Further controls for onset energy, based on correlating loudness at onset with the various eye tracking measures, are described in the Results section.

Participants listened passively to the sounds, which were presented in random order, with a randomized intertrial interval between 6 and 7 s. In total, 320 trials (16 sound-tokens × 20 repetitions of each) were presented. Stimuli were diotically delivered to participants' ears using Sennheiser HD558 headphones via a Creative Sound Blaster X-Fi sound card (Creative Technology) at a comfortable listening level self-adjusted by each participant. Stimulus presentation and response recording were controlled with the Psychtoolbox package (Psychophysics Toolbox Version 3; Brainard, 1997) on MATLAB (The MathWorks R2018a). Participants sat with their head fixed on a chinrest in front of a monitor (viewing distance 65 cm) in a dimly lit and acoustically shielded room (IAC triple-walled sound-attenuating booth). They were instructed to continuously fixate at a black cross presented at the center of the screen against a gray background and to passively listen to the sounds (no task was performed). A 24-inch monitor (BENQ XL2420T) with 1920 × 1080 pixel resolution and 60 Hz refresh rate presented the fixation cross and feedback. The visual display remained the same throughout. To avoid pupillary light reflex effects, display and ambient room luminance were kept constant throughout the experiment. To reduce fatigue, the experiment was divided into 9 4 min blocks, each separated by a 4 min rest period.

##### Pupil measurement.

An infrared eye-tracking camera (Eyelink 1000 Desktop Mount, SR Research) positioned just below the monitor continuously tracked gaze position and recorded pupil diameter, focusing binocularly with a sampling rate of 1000 Hz. The standard five-point calibration procedure for the Eyelink system was conducted before each experimental block. Participants were instructed to blink naturally. They were also encouraged to rest their eyes briefly during intertrial intervals. Before each trial, the eye tracker automatically checked that the participants” eyes were open and fixated appropriately; trials would not start unless this was confirmed.

##### Analysis of eye blinks.

Eye blinks are commonly observed as an involuntary response to abrupt sounds, part of the brainstem-mediated startle reflex (Davis, 1984; Blumenthal and Goode, 1991; Knudson and Melcher, 2016). The elicitation of blinks has been shown to be sensitive to a range of stimulus manipulations (Blumenthal, 2015, 1988) and it was, therefore, important to relate eye-blink incidence to the measures of salience used here.

**Figure 4.**
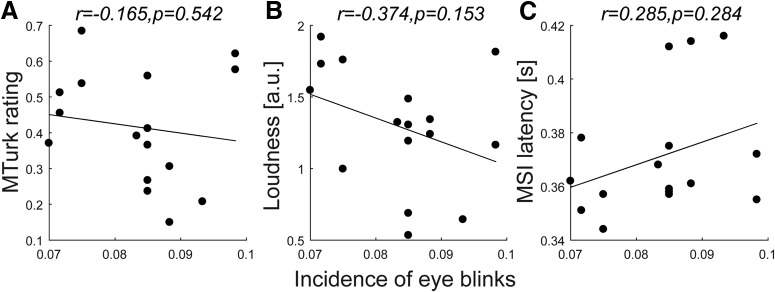
The incidence of eye blinks (i.e., the proportion of trials containing any blinks in the initial 500 ms after sound onset per condition across subjects). The blink rate was not correlated with the crowd-sourced salience rating (***A***), loudness (***B***), or MSI latency (***C***). All correlations are conducted using the Spearman rank method.

Because the blink reflex occurs rapidly after stimulus presentation (Blumenthal, 1988), we analyzed data from the first 500 ms after sound onset. For each subject and sound token, eye-blink incidence was computed by tallying the number of trials that contained a blink (defined as full or partial eye closure). Although the incidence of blinks was low overall (<10%), it varied substantially across participants. For the correlation analyses reported below (Fig. 4), the average rate across participants was computed for each sound condition.

##### Analysis of pupil diameter data.

**Figure 5.**
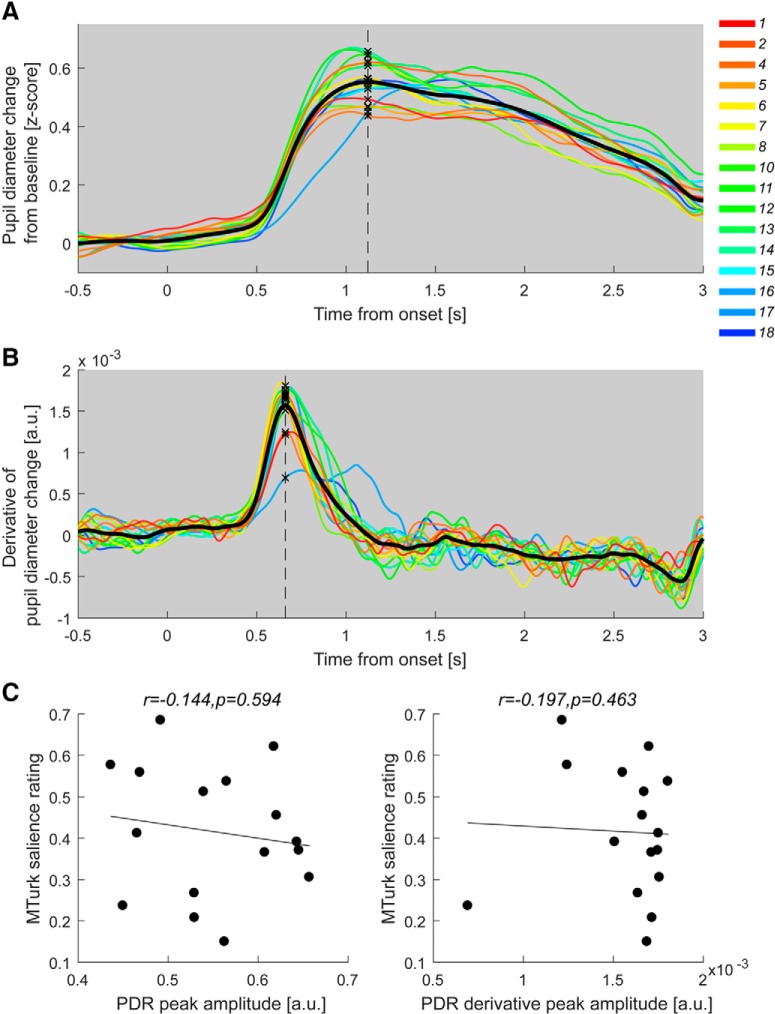
Measures of pupillary dilation are not correlated with crowd-sourced salience. ***A***, The PDR obtained from the full group (*N* = 30). The solid lines represent the average normalized pupil diameter as a function of time relative to the onset of the sound. The line color indicates the MTurk salience ranking; more salient sounds are labeled in increasingly warmer colors. The solid black line is the grand-average across all conditions. The dashed line marks the peak average PDR. ***B***, The PDR derivative. ***C***, Correlations between crowd-sourced salience and peak PDR amplitude (left) and maximum of PDR derivative (right). None of the effects were significant. A similar analysis based on sound-token specific peaks (as opposed to based on the grand average) also did not yield significant effects. All correlations are conducted using the Spearman rank method.

To measure the sound-evoked pupil dilation responses (Fig. 5), the pupil diameter data of each trial were epoched from 0.5 s before to 3 s after sound onset. Intervals where the eye tracker detected full or partial eye closure (manifested as loss of the pupil signal) were automatically treated as missing data and recovered with shape-preserving piecewise cubic interpolation; epochs with >50% missing data were excluded from analysis. On average, less than two trials per participant were rejected.

To compare results across blocks, conditions, and participants, the epoched data within each block were *z*-score normalized. A baseline correction was then applied by subtracting the median pupil size over the pre-onset period; subsequently, data were smoothed with a 150 ms Hanning window.

##### Microsaccade analysis.

Microsaccade detection was based on the algorithm proposed by Engbert and Kliegl (2003). In short, microsaccades were extracted from the continuous horizontal eye-movement data based on the following criteria: (a) a velocity threshold of λ = 6 times the median-based standard deviation within each block; (b) above-threshold velocity lasting for longer than 5 ms but <100 ms; (c) the events are binocular (detected in both eyes) with onset disparity <10 ms; and (d) the interval between successive microsaccades is longer than 50 ms.

Extracted microsaccade events were represented as unit pulses (Dirac delta). Two complementary analysis approaches were employed. The first involved tallying MS events, collapsed across subjects and trials (for more details, see the Results section). The second approach entailed analyzing MS rate time series: For each sound token in each participant, the event time series were summed and normalized by the number of trials and the sampling rate. Then, a causal smoothing kernel, ω(τ) = α^2^ × τ × *e*^−ατ^, was applied with a decay parameter of α = 1/50 ms (Dayan and Abbott, 2001; Rolfs et al., 2008; Widmann et al., 2014), paralleling a similar technique for computing neural firing rates from neuronal spike trains (Dayan and Abbott, 2001; Rolfs et al., 2008; see also Joshi et al., 2016). The obtained time series was then baseline corrected over the pre-onset interval. Due to the low baseline incidence of microsaccades per participant (approximately two events per second) and the small number of presentations per sound token (*n* = 20; required to prevent perceptual adaptation) a within-subject analysis was not possible. Mean microsaccade rate time series, obtained by averaging across participants for each sound token, are used for the analyses reported here. Robustness is verified using bootstrap resampling (see results).

##### Correlation analysis.

To control for outlier effects, all reported bivariate and partial correlations were performed using the conservative Spearman's rank correlation method (two-tailed). The one exception to this was the direct comparison of MSI- and PDR-based correlations, where we computed Pearson correlations between crowd-sourced salience and the various eye tracking measures discussed in the results (MSI latency, PDR peak amplitude, PDR derivative peak amplitude; see defined below). Differences in Pearson correlation coefficients were tested using the procedures for testing statistical differences between correlations using the implementation in the R package cocor (Diedenhofen and Musch, 2015).

## Results

### Crowd-sourced salience ranking yielded a meaningful and stable salience scale

Eighteen environmental sounds (Fig. 1*A* for spectrograms and Fig. 1-1 in the Extended data section for sound files) originally used by Cummings et al. (2006), were selected for this study. The stimulus set represents the variety of sounds that may be encountered in an urban acoustic environment, including animal sounds, human nonspeech sounds (kiss, sneeze), musical instrumental sounds, impact sounds (golf ball, tennis, impact, coins), and an assortment of mechanical sounds (car alarm, alarm clock, camera shutter, pneumatic drill, lawn mower etc.). All stimuli were 500 ms long and RMS equated.

We obtained salience rankings of this sound set from 911 online participants via the Amazon Mechanical Turk (MTurk) platform (see Materials and Methods). Sounds were presented in pairs, and participants were required to report which one was “more salient or attention-grabbing.”

Over all responses, a small but robust order-of-presentation bias for selecting the second sound in a pair was observed (*t* = −9.240, *p* < 0.001; mean probability to choose the first sound = 0.47, mean probability to choose the second sound = 0.53). However, because the order of presentation was counterbalanced across pairs, this bias did not affect the rating results.

To derive a relative measure of salience for each sound, we counted the proportion of pairs (collapsed across all data from all participants) on which each sound was judged as more salient, producing a measure of relative salience ranging between 0 and 1 (Fig. 1*B*). It is striking that a clear scale of subjective salience can be captured across these 18 brief, arbitrarily selected sounds. Variability was estimated using bootstrap resampling (1000 iterations), where on each iteration, salience was computed over a subset of the data (see Materials and Methods). The error bars in Figure 1*B* are 1 SD from this analysis.

### Crowd-sourced salience scale is strongly correlated with in-laboratory salience judgements

An in-laboratory replication was conducted to validate the salience scale obtained from the MTurk experiment. The paradigm was essentially identical, but the experiments were performed in a laboratory setting and under a controlled listening environment. The main differences were that, to reduce test time, the sound set was reduced to 13 sounds (selected to capture the salience range of the full set and indicated in orange bars; Fig. 1*B*) and all participants listened to all sound pairs during an hour-long session.

Because the “in-laboratory” experiment was designed to mirror the MTurk experiment, the planned analysis involved collapsing across trials and subjects in the same way as described above. The in-laboratory ranking showed a strong correlation with the online ranking (*r* = 0.857, *p* < 0.0001; Fig. 1*C*).

The small number of trials per sound-pair (*n* = 4), which was necessary to reduce perceptual adaptation (and to fit into time constraints), produces rather noisy subject-level data. Regardless, we attempted to assess the association between MTurk and in-laboratory rating on an individual level using a repeated measures correlation analysis (rmcorr package in R; Bakdash and Marusich, 2017). The results confirmed that subject level correlation with the MTurk data was significant (Pearson *r* = 0.428, *p* < 0.0001; Spearman *r* = 0.455, *p* < 0.0001), though, as expected, accounting for noise at the individual subject level resulted in a decreased explained variance. Overall, the individual level analysis supports the conclusion that the group level data can be taken as representative of single subjects.

### Crowd-sourced salience correlates with acoustic roughness

Although the present set of sounds is too small to systematically pinpoint the sound features that contribute to auditory salience, we sought to understand whether the obtained “subjective” salience scale correlates with several key acoustic features, previously hypothesized as contributing to salience:

We found that, despite the fact that loudness is known to be a prominent contributor to perceptual salience (Kayser et al., 2005; Liao et al., 2016; Huang and Elhilali, 2017), the crowd-sourced salience scale in the present set did not significantly correlate with loudness (*r* = 0.428, *p* = 0.078; see Materials and Methods for details about the loudness measure). This may be partly because the level of these sounds was RMS-normalized thus removing some of the larger differences in loudness between sounds.

Next, we tested the relationship between crowd-sourced salience and measures of salience derived from the model of Kayser et al. (2005). Several relevant parameters were examined (see Materials and Methods). Only correlations with the gradient along the frequency dimension were significant (Spearman's *r* = 0.525 *p* = 0.027 for the maximum gradient and *r* = 0.488, *p* = 0.049 for the mean gradient; for the rest of the comparisons *p* ≥ 0.155), indicating that perceptual salience may be associated with salience maps in which salient regions are sparsely spread across the spectrum.

Motivated by previous work (Sato et al., 2007; Arnal et al., 2015; Huang and Elhilali, 2017), we also investigated the correlation between perceptual salience and roughness -a perceptual quality that is associated with energy in the high end (>30 Hz) of the amplitude modulation spectrum (e.g., Arnal et al., 2015; see Materials and Methods). Here, the correlation between crowd-sourced salience and roughness yielded a significant effect (*r* = 0.709, *p* = 0.001; Fig. 1*D*), consistent with accumulating evidence that roughness is a major contributor to salience.

### Crowd-sourced salience correlates with objective measures from ocular dynamics

Next, we investigated whether acoustic salience automatically (i.e., without a remit from a task) modulates ocular orienting responses. A subset of 16 of the 18 original sounds (two sounds, 3 and 9, were excluded due to experimental time constraints) were presented to naive, centrally-fixating subjects who listened passively to the sounds, without performing any task, while their gaze position and pupil diameter were continuously tracked. Sounds were presented in random order, and with a random intersound interval between 6 and 7 s. Overall, each sound was presented 20 times across the experimental session. This small number of repetitions was chosen so as to minimize potential effects of perceptual adaptation to the stimuli. The analysis is therefore based on group-level correlations. Resampling based analyses were conducted to derive an estimate of the distribution of correlation strengths in the population.

We analyzed two types of rapid orienting responses: the “ocular freezing” (MSI) response (Hafed and Clark, 2002; Engbert and Kliegl, 2003; Rolfs et al., 2008; Hafed and Ignashchenkova, 2013) and the PDR (Wang and Munoz, 2015; Wang et al., 2017). We also analyzed the incidence of eye blinks and their possible relationship to perceptual salience. Eye blinks are a component of the brainstem-mediated startle reflex (Davis, 1984), hypothesized to reflect an automatic defensive response to abrupt or threatening stimuli. The startle eye blink response is commonly elicited by loud, rapidly rising sounds (Blumenthal and Goode, 1991; Knudson and Melcher, 2016), but has been shown to be sensitive to a range of stimulus manipulations (Blumenthal, 2015, 1988).

#### Incidence of eye blinks was not correlated with crowd-sourced salience

The incidence of eye blinks was low overall (<10%) and did not significantly correlate with any of the measures reported here.

#### Measures of pupillary dilation were not correlated with crowd-sourced salience

The temporal evolution of the normalized pupil diameter (the pupil dilation response, PDR) is presented in Figure 5. The pupil starts to dilate around 0.5 s after sound onset, and peaks at ∼1.12 s (ranging from 1.02 to 1.33 s). We did not observe significant correlation between crowd-sourced salience rating and any key parameters associated with PDR dynamics (see Fig. 5*C* for statistics), including the PDR peak amplitude and the peak of the PDR derivative (maximum rate of change of the PDR).

#### Crowd-sourced salience is correlated with MSI

**Figure 6.**
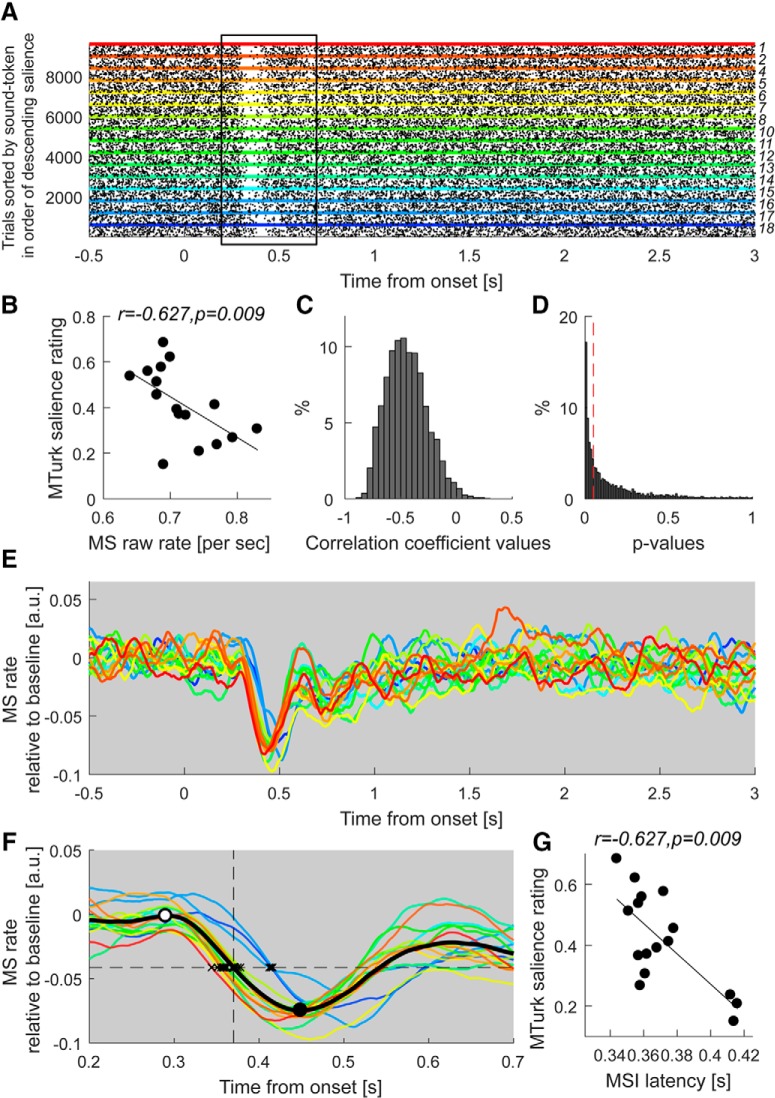
MSI is correlated with crowd-sourced salience. ***A***, Raster plot of microsaccade events (pooled across all participants) as a function of time relative to sound onset. The *y*-axis represents single trials; each dot indicates the onset of a microsaccade. Trials are grouped by sound-token and arranged according to the MTurk-derived salience scale (increasingly hot colors indicate rising salience). The region of MSI, between 0.2 and 0.7 s post-sound onset, is highlighted with a black rectangle. ***B***, Over this time interval, the MS rate (number of MS events per second) is correlated significantly with the crowd-sourced salience rating. The result of bootstrap resampling is shown as the distribution of correlation coefficients (***C***) and the distribution of associated *p*-values (***D***). The vertical red dashed line indicates *p* = 0.05. ***E***, Average microsaccade rate time series for each sound (***F***) focusing on the MSI region. The solid black curve is the grand-average MS rate across all sound tokens. MSI commences at ∼0.3 s after sound onset (open circle) and peaks around 0.45 s (solid black circle). The horizontal dashed line indicates the mid-slope of the grand average (amplitude = −0.04 a.u., time = 0.37 s). Black crosses mark the time at which the response to each sound intersects with this line, as a measure of MSI latency. ***G***, Correlation between these values and the crowd-sourced salience rating. All correlations are conducted using the Spearman rank method. Note identical correlation values in ***G*** and ***B*** are a chance occurrence (the two analyses are independent).

The microsaccade results are shown in Figure 6. Consistent with previous demonstrations (Rolfs et al., 2008), we observed an abrupt inhibition of microsaccadic activity after sound presentation. The drop in microsaccade rate began at ∼0.3 s after onset and reached a minimum at 0.45 s.

We conducted two different analyses to determine the extent to which MSI differs across sounds. The first approach is based on pooling MS data across trials and subjects and counting MS events. We defined a window spanning a 500 ms interval from 200 to 700 ms after sound onset. This window encompasses the interval before the beginning of MSI and after it has settled (Fig. 6*A*; the extent of the interval is also shown in Fig. 6*F*). We then tallied the MS events for each sound token. This measure correlated with crowd-sourced salience such that more salient sounds were associated with fewer MS events (i.e., a lower MS rate) within the window (*r* = −0.627, *p* = 0.009; Fig. 6*B*). As can be seen in Figure 6*B* the number of MS events is small overall and the differences between conditions are narrow, reflecting the low incidence of micro saccades. The robustness of the observed correlation was confirmed with bootstrap resampling (see Fig. 6*C*; 5000 iterations; balanced) where on each iteration we selected 30 participants with replacement to compute the tally. This analysis (Fig. 6*C*) confirmed a negatively skewed distribution of r values centered around −0.5 (median *r* = −0.458), with 98.72% of r values smaller than 0 (*p* = 0.013) and a left skewed distribution of associated *p*-values (Fig. 6*D*). This effect was maintained for windows spanning up to 1 s from sound onset.

The MS rate demonstrated a significant correlation with roughness (*r* = −0.607 *p* = 0.013) but not with loudness (r-0.353 *p* = 0.18). The correlation between MS rate and crowd-sourced salience was no longer significant when controlling for roughness as a covariate (partial correlation *r*_(13)_ = −0.350 *p* = 0.201), suggesting that dependence on roughness is a major contributor to the correlation between MSI and crowd-sourced salience.

Next, we aimed to understand more precisely how the dynamics of MSI vary with salience by quantifying the MSI latency for each sound. This was accomplished by computing a MS rate time series for each token (Fig. 6*E*; see Materials and Methods). MSI latency was then determined by computing a grand-mean microsaccade rate time series (averaged across sound tokens; see Fig. 6*E*), identifying its mid-slope amplitude (horizontal dashed line in Fig. 6*F*), and obtaining the time at which the microsaccade rate time series associated with each sound token intersected with this value. This latency, hereafter referred to as the MSI latency, correlated with the crowd-sourced salience rating (*r* = −0.627, *p* = 0.009; Fig. 6*G*), such that increasing salience was associated with earlier MSI.

The correlation between MSI latency and crowd-sourced salience was significantly different from the PDR correlations with crowd-sourced salience reported above (MSI vs PDR: *z* = 2.6369, *p* = 0.0042; MSI vs PDR derivative: *z* = 3.0640, *p* = 0.0011; see Materials and Methods). It was further confirmed that MSI latency did not significantly correlate with blink rates within the first 500 ms of sound onset (Fig. 4*C*).

**Figure 7.**
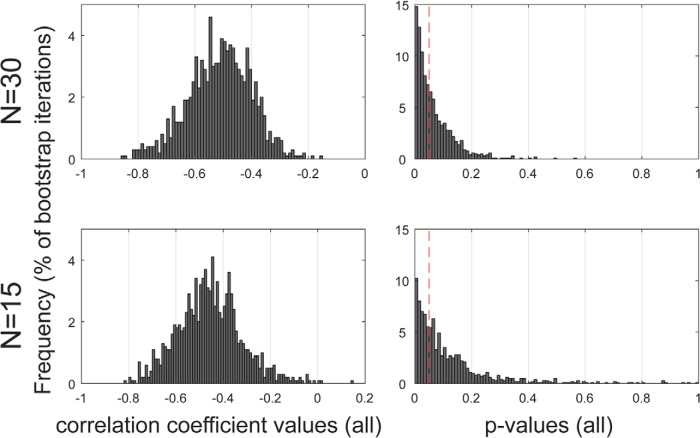
Estimation of the stability of the correlation between MSI latency and crowd-sourced salience. Left, Distribution of the Spearman correlation coefficients derived from resampling analyses with subgroup sizes of 15 or 30 participants. In both cases, the distribution peaks around *r* = −0.5. Right, Distribution of the *p*-values associated with each *n*. The red vertical dashed line indicates *p* = 0.05. A uniform distribution is expected under the null. The left skewed pattern observed here indicates a true effect. Skewness was formally confirmed by a χ^2^ test on *p*-values <0.05 (*n* = 30: χ^2^(1066) = 1405.42, *p* < 0.0001; *n* = 15: χ^2^(748) = 903.2, *p* < 0.0001).

Additional analyses to confirm effect robustness (Fig. 7) used bootstrap resampling to estimate the stability of the correlation between MSI latency and crowd-sourced salience across the subject pool. This involved computing a distribution of *p*- and *r*-values for subgroup sizes of 30 and 15 subjects (with replacement). We iteratively (5000 iterations) selected *n* samples (*n* = 15 or 30) from the full pool of *N* = 30. For each subset, we computed the correlation between MSI latency and crowd-sourced salience. The distribution of associated correlation coefficients demonstrated a moderate correlation (median *r* = −0.5063 for N = 15, *r* = −0.4615 for *N* = 30) between MSI latency and crowd-sourced salience. The distributions of *p*-values are significantly left-skewed (Fisher's method; *p* < 0.0001; further details in the figure), indicative of a true effect.

To determine what acoustic information might have driven the observed microsaccade effect, we correlated the MSI latency with the measures obtained from the Kayser et al. (2005) model (see Materials and Methods). This analysis revealed no significant correlations (*p* ≥ 0.203 for all).

We also correlated MSI latency for each sound with roughness and loudness estimates computed between 0 and 300 ms (window sizes of 50, 100, 150, 200, 250 and 300 ms) - e.g., over the interval between sound onset and the average onset time of ocular inhibition. For loudness, none of the correlations reached significance (*p* > 0.152), This suggests that though the sounds used had clearly differing distributions of power at onset, this did not contribute primarily to the correlation with MSI. The correlation between MSI latency and crowd-sourced salience was maintained even when controlling for loudness at onset (0–50 ms window from onset; partial correlation; *r*_(13)_ = −0.666, *p* = 0.007; same holds for longer intervals).

In contrast to the lack of a stable link between loudness and MSI latency, a significant correlation with roughness was present from 250 ms onwards (*p* ≤ 0.028, *r* ≥ −0.547), confirming the previous observations of a strong link between roughness and MSI rate. The correlation between MSI latency and salience was no longer significant when controlling for roughness as a covariate (partial correlation *r*_(13)_ = −0.455 *p* = 0.088), suggesting that dependence on roughness is a major contributor to the correlation between MSI and crowd-sourced salience.

## Discussion

The main aim of this work was to understand whether/how ocular orienting responses in naive listeners are modulated by acoustic salience. We showed that a crowd-sourced 'subjective” (i.e., rating based) salience ranking of brief, nonspatial, environmental sounds robustly correlated with the ocular freezing response measured in naive, passively listening participants. Sounds ranked by a large group of online participants as more salient evoked earlier MSI, consistent with a faster orienting response (Fig. 6). These results establish that information about auditory salience is conveyed to the SC, the primary generator of micro saccades, within ∼300 ms after sound onset. That sounds systematically modulated microsaccade activity demonstrates that the mechanisms that drive MSI are sensitive to a broad range of salient events beyond the visual modality.

### Crowd-sourced salience

We demonstrated that a robust measure of perceptual salience can be obtained from a web-based mass-participant experimental platform. Online experimenting is gaining popularity within cognitive science (for review, see Stewart et al., 2017), including in the auditory modality (Woods et al., 2017; Woods and McDermott, 2018). However, there are various potential drawbacks to this approach relating to lack of control over the participants” listening devices and environment. These may be especially severe for perceptual judgment experiments that demand a high level of engagement from participants. However, the limitations are offset by important unique advantages, including the opportunity of obtaining a large amount of data in a short period of time, and running brief “one-shot” experiments that are critical for avoiding perceptual adaptation. Furthermore, in the context of salience, the variability of the sound environment may in fact provide “real-world” validity to the obtained scale. Here, we established that despite the various concerns outlined above, capitalizing on big numbers makes it possible to acquire a stable, informative, salience scale with relatively minimal control of the listeners and their environment. Indeed, the salience scale obtained online correlated robustly with in-laboratory ranking measures as well as with certain acoustic features previously established as contributing to perceptual salience.

Specifically, we found a strong correlation with “roughness,” the perceptual attribute that is associated with “raspy,” “buzzing,” or “harsh” sounds. This correlation arose “organically” in the sense that the sounds in the present study were not selected to vary across this or other acoustic dimensions. The link between roughness and salience is consistent with previous reports (Sato et al., 2007; Arnal et al., 2015; Huang and Elhilali, 2017) establishing a clear role for this feature in determining the perceptual prominence of sounds. Most recently, this was demonstrated in the context of the distinctiveness of screams (Arnal et al., 2015; though the authors used the term “fearful” as opposed to “salient” in their experiments).

Kayser et al. (2005) have proposed a model for auditory salience, inspired in its architecture by the well established model for visual salience (Itti and Koch, 2001). We found limited correlation between the parameters derived from that model and the present crowd-sourced scale. This is possibly because the Kayser model is better suited to capturing “pop-out”-like saliency, associated with attentional capture by an object that stands out from its background. Instead, here we focused on brief sounds reflecting single acoustic sources.

It is important to stress that the present sound set is too small for an extensive exploration of the features that might drive perceptual salience. Roughness likely stood out here because of the primacy of that feature and because our sounds spanned a large enough roughness range (Fig. 3). The robustness of the crowd-sourced judgements suggests that a similar crowd-sourcing approach but with a larger, and perhaps more controlled, set of sounds may reveal other relevant sound features. In particular, recent advances in sound synthesis technologies make it possible to systematically and independently vary acoustic features toward a controlled investigation of acoustic salience.

### Acoustic salience did not modulate pupil responses

The PDR indexes activity within the LC-norepinephrine system (Aston-Jones and Cohen, 2005; Joshi et al., 2016), which is proposed to play a key role in controlling global vigilance and arousal (Sara, 2009; Sara and Bouret, 2012). In previous work reporting an association between the PDR and sound salience, the dominant driving feature for the correlation was loudness (Liao et al., 2016; Huang and Elhilali, 2017). In contrast, differences along this dimension were minimized in the present stimuli to allow us to focus on subtler, but potentially behaviorally important, contributors to perceptual salience. Our failure to observe a modulation of the PDR by salience suggests that, at least in the context of auditory inputs, pupil dilation may reflect a nonspecific arousal response, evoked by stimuli that cross a certain salience threshold. This account is consistent with the relatively late timing of the PDR (peaking approximately 1 s after sound onset) thereby potentially reflecting a later stage of processing than that captured by microsaccades (see below).

### MSI is a correlate of acoustic salience

We revealed a robust correlation between MSI latency and crowd-sourced salience: Sounds judged by online raters as more salient were associated with a more rapid (Fig. 6*G*) and extensive (as reflected by decreased incidence; Fig. 6*B*) inhibition of microsaccadic activity. The effect arose early, from ∼350 ms after sound onset, pointing to fast underlying circuitry. Correlation analyses indicated that the bulk of this effect is driven by a correlation with roughness, suggesting that this information is computed sufficiently early to affect the body's automatic reorienting response.

The brain mechanisms that respond to acoustic roughness are poorly understood. Response signatures have been observed in both auditory cortical and subcortical areas (Schnupp et al., 2015), including the amygdala, a key brain center for fear/risk processing (Adolphs et al., 1995; Nader et al., 2000; Bach et al., 2008; Ciocchi et al., 2010; Arnal et al., 2015). Arnal et al. (2015) reported that the amygdala, but not auditory cortex, exhibited specific sensitivity to temporal modulations within the roughness range. This was interpreted as suggesting that rough sounds activate neural systems associated with the processing of danger. The present findings, demonstrating an association between salience/roughness and rapid orienting responses, are consistent with this conclusion.

Microsaccades are increasingly understood to index an active attentional sampling mechanism that is mediated by the SC (Rolfs, 2009; Hafed et al., 2015; Rucci and Poletti, 2015; Wang and Munoz, 2015; Krauzlis et al., 2018). Accumulating work suggests that MS occurrence is not automatic but rather modulated by the general state of the participant and by the availability of computational capacity, such that microsaccade incidence is reduced under high load (Widmann et al., 2014; Gao et al., 2015; Dalmaso et al., 2017; Yablonski et al., 2017). MSI is an extreme case for such an effect of attentional capture on ocular dynamics, interpreted as reflecting an interruption of ongoing attentional sampling so as to prioritize the processing of a potentially important sensory event. The dominant account for MSI is that sensory input to the SC causes an interruption to ongoing activity by disturbing the balance of inhibition and excitation (Rolfs et al., 2008). Previously reported effects of visual salience on MSI (Rolfs et al., 2008; Bonneh et al., 2015; Wang et al., 2017) were therefore interpreted as indicating that visual salience may be coded at the level of the SC (see also Mizzi and Michael, 2014; Veale et al., 2017; White et al., 2017a,b). We showed that the perceptual salience of sounds also modulates this response, consistent with a well-established role for the SC as a multisensory hub (Meredith and Stein, 1986; Meredith et al., 1987; Wallace et al., 1998; Wang et al., 2017). Importantly, this effect was observed during diotic presentation—sounds did not differ spatially and were perceived centrally, within the head.

The present results thus suggest that an investigation of SC responses to sound may provide important clues to understanding auditory salience. There is evidence for projections from the auditory cortex to the SC (Meredith and Clemo, 1989; Zingg et al., 2017) that might mediate the effects observed here, or they may arise via a subcortical pathway with the IC (Xiong et al., 2015) or the amygdala as an intermediary.

Finally, the present experiments focused on the salience of brief sounds presented in silence. However, the ongoing context within which sounds are presented is known to play a critical role in determining their perceptual distinctiveness (Leech et al., 2007; Krishnan et al., 2013; Kaya and Elhilali, 2014; Sohoglu and Chait, 2016; Southwell and Chait, 2018). In the future, the paradigm established here can be easily expanded to more complex figure–ground situations or to tracking salience within realistic sound mixtures. A further question relates to understanding whether ocular dynamics reflect perceptual salience primarily linked to basic, evolutionary-driven sound features such as roughness or whether they can also be modulated by arbitrary sounds endowed with salience via association or reinforcement (e.g., ones' mobile ring tone).
