# Supplementary figures and images for: Rapid Ocular Responses Are Modulated by Bottom-up-Driven Auditory Salience
Source: J Neurosci. 2019 Sep 25;39(39):7703–14. doi: 10.1523/JNEUROSCI.0776-19.2019 (PMC6764203; doi:10.1523/JNEUROSCI.0776-19.2019)

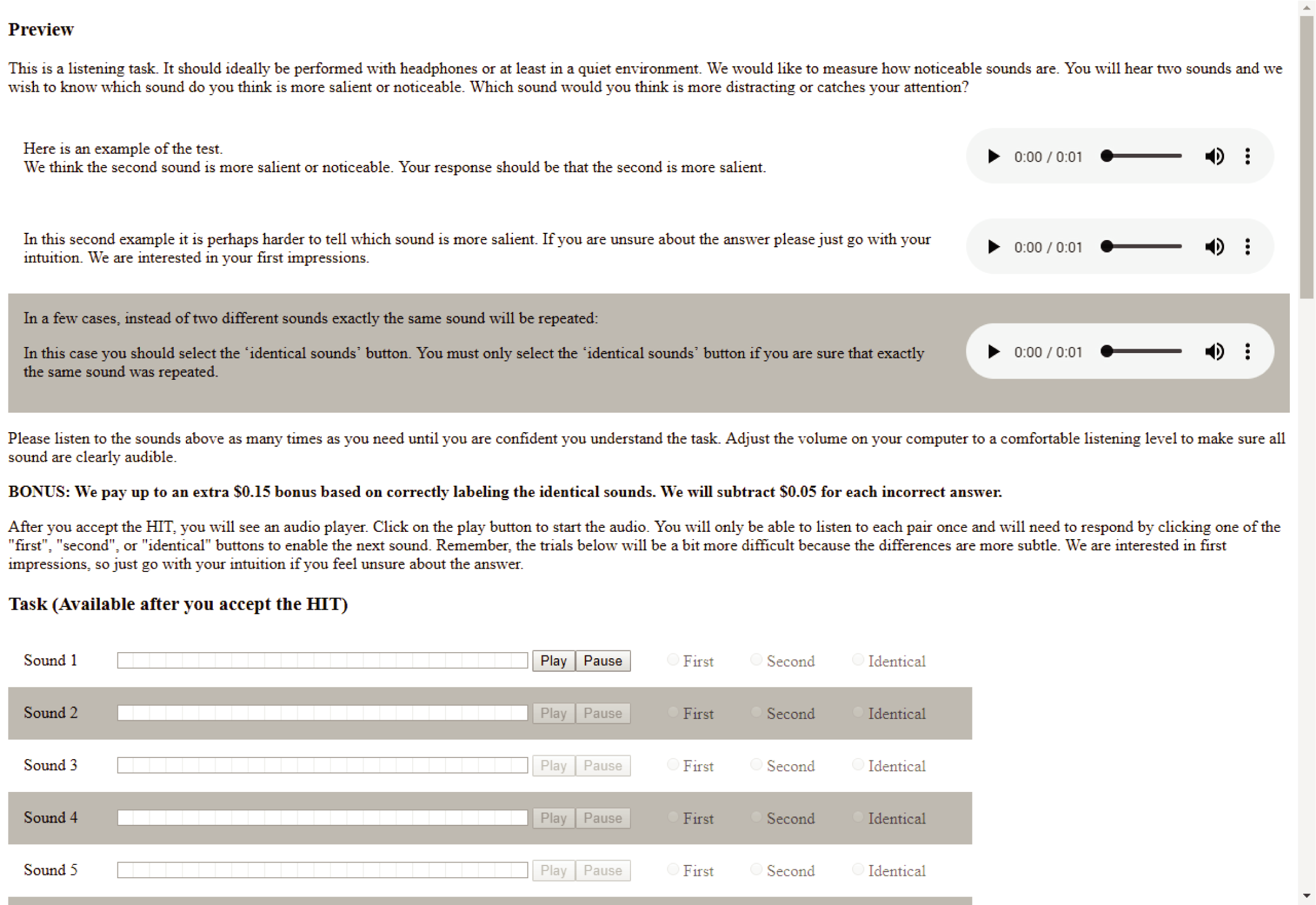

Supplement: Figure 1-2 [file zns999191931so18.tif]
